# The Role of Dietary Fat throughout the Prostate Cancer Trajectory

**DOI:** 10.3390/nu6126095

**Published:** 2014-12-22

**Authors:** Katie M. Di Sebastiano, Marina Mourtzakis

**Affiliations:** Department of Kinesiology, University of Waterloo, 200 University Avenue W., Waterloo, ON N2L 3G1, Canada; E-Mail: kmdiseba@uwaterloo.ca

**Keywords:** risk, progression, survivorship, IGF signaling, saturated fatty acids, monounsaturated fatty acids, polyunsaturated fatty acids, trans fatty acids

## Abstract

Prostate cancer is the second most common cancer diagnosed world-wide; however, patients demonstrate exceptionally high survival rates. Many lifestyle factors, including obesity and diet, are considered risk factors for advanced prostate cancer. Dietary fat is a fundamental contributor to obesity and may be specifically important for prostate cancer patients. Prostate cancer treatment can result in changes in body composition, affecting quality of life for survivors by increasing the risk of co-morbidities, like cardiovascular disease and diabetes. We aim to examine dietary fat throughout the prostate cancer treatment trajectory, including risk, cancer development and survivorship. Focusing on one specific nutrient throughout the prostate cancer trajectory provides a unique perspective of dietary fat in prostate cancer and the mechanisms that may exacerbate prostate cancer risk, progression and recurrence. Through this approach, we noted that high intake of dietary fat, especially, high intake of animal and saturated fats, may be associated with increased prostate cancer risk. In contrast, a low-fat diet, specifically low in saturated fat, may be beneficial for prostate cancer survivors by reducing tumor angiogenesis and cancer recurrence. The insulin-like growth factor (IGF)/Akt signaling pathway appears to be the key pathway moderating dietary fat intake and prostate cancer development and progression.

## 1. Introduction

Prostate cancer is the second most commonly diagnosed malignancy in men worldwide. Prostate cancer diagnoses accounts for 15% of all male cancer diagnoses, second to only lung cancer [1,2]. In 2012, more than 1.1 million prostate cancer cases were diagnosed worldwide [1,2]; however, prostate cancer mortality rates are exceptionally low, with only 307,000 prostate cancer deaths estimated in 2012, accounting for only 6.6% of male deaths. [1]. This remarkably low mortality rate is attributed to the wide-spread use of prostate-specific antigen (PSA) screening in most developed countries where the incidence of prostate cancer is higher. PSA screening allows the detection of smaller and earlier stage tumors that may or may not progress to more advanced cancer. Because of the growing incidence of prostate cancer and the low death rates, prevention of prostate cancer and, specifically, aggressive and advanced prostate cancer is of the utmost importance. Many lifestyle factors, such as obesity, diet, physical activity levels and smoking, are considered risk factors in the development of prostate cancer. Consequently, a large body of literature endeavours to elucidate the role of lifestyle factors, including obesity, in prostate cancer risk, development of the tumour and successful survivorship.

Obesity and prostate cancer endure a complex relationship. Obesity is associated with increased incidence of high-risk or aggressive prostate cancer [3]. Additionally, it is associated with increased incidence of prostate cancer recurrence [4], which may be attributed to increased adiposity and reduced muscularity in prostate cancer patients who undergo androgen deprivation therapy (ADT) [5]. One of the potential factors thought to link obesity and prostate cancer is dietary fat intake. Dietary fat is a fundamental contributor to obesity [6] and may help explain the complicated relationship between obesity and prostate cancer. For prostate cancer patients, obesity is not only a potential risk factor, but changes in body composition may affect the quality of life of prostate cancer survivors by increasing the risk of co-morbidities, like cardiovascular disease [7], and by decreasing functional outcomes [8]. Changes in dietary fat intake may help ameliorate some of the negative outcomes associated with changes in body composition in the prostate cancer survivor. Thus, many studies have looked at diet and exercise manipulation to counter the expected increase in adiposity and the loss of muscle. Further consideration into the role of obesity, dietary fat and the prostate cancer trajectory is warranted to help clarify this complex relationship.

The aim of this manuscript is to review the literature examining dietary fat throughout the prostate cancer trajectory, including risk, development and survivorship. The majority of reviews that examine the role of dietary fat in prostate cancer consider many nutrients at one particular time point during the prostate cancer trajectory (*i.e.*, the role of diet in prostate cancer risk). This narrative review will examine and summarize the role of dietary fat in prostate cancer throughout the trajectory to provide a unique and comparative perspective across the time-course of the disease. We also aim to investigate the mechanisms that exacerbate prostate cancer risk, progression and recurrence and how they may be related. Saturated fat, *n*-3 fatty acids and trans-fatty acids will be given special considerations, as these types of dietary fat may be of particular importance for the prostate cancer patient. The perspectives described in this review will facilitate the identification of gaps in the literature and will aid future studies to advance this discipline.

## 2. Dietary Fat and Prostate Cancer Risk

There are numerous nutritional factors associated with obesity and prostate cancer risk, including positive energy balance [9], red meat and dairy intake [10], saturated fat [11], trans fatty acid intake [12] and total dietary fat intake [11]. Conversely, *n*-3 fatty acids have been identified as having a potentially protective effect against prostate cancer [11]. The earliest identification of dietary fat as a potential risk factor for prostate cancer stems from correlations and case-controls studies; however, the results were mixed, with studies demonstrating positive, negative and no associations between dietary fat and the risk of prostate cancer. These studies are based on the principle that participants moving from countries where the risk of prostate cancer is low, such as Japan, had a significant increase in the risk of developing prostate cancer upon moving to North America [13,14]. These studies hypothesized that increased dietary fat consumption may be driving this relationship [15]; however, they did not quantify dietary fat, nor did they control for confounders, which may affect the association with prostate cancer. Early case-control studies examining dietary fat as a risk factor for prostate cancer also demonstrated mixed results [16,17,18,19,20,21,22,23]. Some of these case-control studies demonstrated positive associations between both dietary and saturated fat intake [16,17,18,19], while others found no association between dietary fat intake and prostate cancer diagnosis [20,21,22,23]. Early prospective cohort studies also demonstrated mixed results, with some demonstrating no association between high meat and dairy products [24,25,26] and prostate cancer risk, while animal product intake was positively associated with the risk of prostate cancer, though these associations were weak (relative risk: 1.3–1.5; *p* > 0.1 [27]; relative risk: 1.38, 95% CI: 0.89–2.16) [27,28]. These studies were also limited by the methods used to assess dietary intake. It was not until Giovannucci and colleagues [29] published their prospective analysis of dietary fat and prostate cancer risk using the Health Professionals follow-up study that the relationship between dietary fat and prostate cancer risk began to become clearer. Giovannucci *et al.* [29] used a semi-quantitative food frequency questionnaire to assess dietary fat in these men. They concluded that total fat consumption was related to the risk of advanced cancer and that this relationship was primarily related to animal fat consumption (relative risk: 1.79; 95% CI: 1.04–3.07). Fat from dairy products, with the exception of butter, appeared to be unrelated to advanced prostate cancer risk.

These early studies formed the basis for much of the current work that has evaluated dietary fat intake as a potential risk factor for prostate cancer. A systematic review by Ma and Chapman [30] indicated that based on the Oxford Centre for Evidenced-Based Medicine Levels of Evidence, there is Level 2b evidence to suggest that dietary fat, and in particular, high intake of animal and saturated fats (~40% total fat intake; [31]), is associated with the increased risk of prostate cancer. Most of the studies evaluated in the systematic review were primarily Level 4 studies (Case series), but also including some Level 2 (prospective comparative) and 3 (retrospective cohort and case-control) studies. Ma and Chapman [30] examined numerous types of fat and identified that there is suggestive evidence to support total fat intake, animal and saturated fat intake, monounsaturated fatty acids and α-linoleic acid as being associated with the increased risk of prostate cancer, while there was not enough evidence to draw conclusions about polyunsaturated fatty acid intake, as well as eicosapentaenoic acid (EPA).

Gathirua-Mwangi and Zhang [32] evaluated the relationship between dietary fat intake and the risk of advanced cancer. Despite the limited number of studies, their conclusions were similar to Ma and Chapman [30], indicating that total fat intake (odds ratios: 1.25–1.80; 95% CI: 0.75–2.91) and, specifically, saturated fat intake are significantly associated with the increased risk of advanced prostate cancer (odds ratios: 1.44–1.80; 95% CI: 0.82–5.20) [32]. When they evaluated the classification of fatty acids, they concluded that monounsaturated and polyunsaturated fatty acids were not associated with the risk of advanced prostate cancer, nor were linoleic and linolenic acids. They also noted that higher animal fat intake was also associated with advanced prostate cancer, and a borderline inverse relationship was noted for total vegetable fat intake and advanced prostate cancer risk. The most recent evidence for this relationship comes from Pelser and colleagues [11]. They suggested that a dichotomous relationship between dietary fat consumption and prostate cancer risk exists. Using data from the NIH-AARP Diet and Health Study, they demonstrated that total fat intake and mono- and poly-unsaturated fats were not associated with the incidence of prostate cancer, but saturated fat increased the risk of advanced prostate cancer (hazards ratio: 1.21; 95% CI: 1.00–1.46) and prostate cancer death (hazards ratio: 1.47; 95% CI: 1.01–2.15). They also noted that EPA was associated with decreased risk of fatal prostate cancer (hazards ratio: 0.82; 95% CI: 0.64–1.04).

Despite the limited number of studies, the systematic reviews cited here indicated some evidence for the increased risk of prostate cancer and, specifically, advanced or fatal prostate cancer with increased dietary fat intake. When evaluating specific categories of fats, saturated and animal fats appear to pose the greatest risk for prostate cancer development, while EPA may have a protective effect. However, more evidence is needed to understand the potential mechanisms that drive the relationship between fat intake and prostate cancer development.

## 3. Effects of Dietary Fat on Prostate Cancer Development

A number of potential mechanisms have been identified that may mediate the effects of dietary fat on prostate cancer development and progression. These include changes in the insulin-like growth factor (IGF)-Akt pathway, androgen signaling and alterations in cell proliferation and angiogenesis.

### 3.1. The IGF Signaling Pathway

The IGF signaling pathway is one of the main regulating pathways in which dietary fat can promote prostate cancer development and progression. Dietary fat intake is positively correlated with circulating levels of IGF-1, which may ultimately result in increased signaling through the IGF signaling pathway [33,34]. Fat intake is also negatively correlated with insulin-like growth factor binding protein-3 (IGFBP-3), the major binding protein of IGF-1 in plasma [33,34], which is also independently associated with the risk of prostate cancer [35]. Thus, increases in circulating levels of IGF-1 and decreases in IFGBP-3 can increase the stimulation of the IGF signaling cascade, which becomes disrupted in malignant cells. Given that perturbations in the IGF system play a critical role in cell proliferation, differentiation, apoptosis and transformation, understanding the function of IGF signaling is key to determining the mechanisms of dietary fat in prostate cancer development and proliferation.

In non-malignant cells, IGF-1 binds to one of two receptors, IGF-1R and IGF-2R, with a preference for IGF-1R [36]. IGF-1R is a type 2 tyrosine kinase receptor that, under normal conditions, is involved in proliferation and differentiation; however, in transformed malignant cells, IGF-1R plays a key role in the establishment and progression of cancerous cells [36]. Cells lines without IGF-1R have an impaired ability to transform into malignant cells, though the exact mechanism is unknown [37]. In contrast, the presence of IGF-1R may also contribute to the ability of malignant cells to metastasize [36]. The primary role of IGF-1R cell growth is mediated through the IGF-1R/insulin receptor substrate (IRS)-1 axis. Numerous reviews on the role of the IGF-1R/IRS-1 axis and its role in cancer are available [36,37,38,39,40,41]. Briefly, when a ligand binds to IGF-1R, it activates the tyrosine kinase of the cytoplasmic domain of the receptor [36]. This results in the phosphorylation of the numerous IGF-1R substrates—IRS-1 and -2 Src- and collagen-homology (SHC) and growth factor receptor protein 2 (Grb2) [38,39,40,41]. The phosphorylated IRS and SHC in combination with Grb2 activate the mitogen-activated protein kinase (MAPK) cascade, resulting in cell growth and proliferation [38,39,40,41]. IRS-1 also phosphorylates phosphatidylinositol 3′-kinase (PI3K) and the Akt complex, which blocks Bad and Caspase 9, which are key pro-apoptotic proteins [38,39,40,41]. These pathways help regulate the metabolic and anti-apoptotic signal of IGF-1 [38,39,40,41]. Akt can also activate the transcription of nuclear factor-κB (NFκB) and mammalian target of rapamycin (mTOR). When NFκB is active in healthy cells, it functions as a regulator for many genes that control proliferation and cell survival; in cancerous cells, it is dysregulated, resulting in decreased cell death [38,39,40,41]. Akt activation promotes signaling in the IGF-1R/IRS-1 axis, which contributes to the dysregulation of NFκB and, ultimately, cancer cell growth. The IGF-1R/IRS-1 axis also indirectly increases mTOR activity [38,39,40,41]. mTOR then promotes cell growth through the S6kinase, Protein Kinase C (PKC), p21 and Glycogen Synthase Kinase 3β (GSK3β) activation [38]. These pathways change the cell cycle, ultimately promoting cell growth. Thus, increased dietary fat intake may potentially promote malignant cell growth through increased IGF-1 and decreased IGFBP-3, resulting in increased IGF-1 signaling through the IGF-1R, a receptor implicated in the transformation of healthy cells to cancerous cells and a mediator of cell growth through the IGF-1R/IRS-1 axis.

### 3.2. Increases in Androgen Signaling Due to Dietary Fat Intake

Along with IGF-1 signaling, androgen signaling is another pathway in which dietary fat intake can influence prostate cancer development. Some have demonstrated that decreased dietary fat intake is associated with decreased androgen [42,43] and testosterone levels [44,45,46], which subsequently improves signaling mediated through the IGF-1 signaling pathway. Androgens play a key role in the development of normal healthy prostate tissue; however, androgen signaling and, specifically, the androgen receptor, also known as nuclear receptor subfamily 3, group C, member 4, (NR3C4), is the principle stimulant of prostate cancer progression. In the early stages of development, malignant prostate cancer cells require androgen stimulation for growth [47]. However, increased androgen receptor growth is associated with the progression or switch of hormone sensitive cancers to hormone-resistant cancers, the more aggressive form of prostate cancer [47,48]. Androgens stimulate prostate cancer cell growth via the Erk-2 pathway, where Erk-2 activation increases the androgen receptor complex content in the prostate cells [49]. Androgens also increase IGF-1R expression [50], which is associated with prostate cancer development, as previously described, but IGF-1 can also have a more direct effect on the androgen receptor. Stimulation of the IGF-1 signaling cascade activates MAPK, which decreases the acetylation of heat shock protein (HSP) 90. HSP90 is a chaperone protein of the androgen receptor [51]. This decreased acetylation increases the association of the HSP90 with the androgen receptor, which further stimulates the signaling through the androgen receptor pathway. Ultimately, stimulation of this pathway results in upregulation of the androgen receptors, their associated proteins and androgen receptor regulated genes. High-fat diets have been shown to increase stimulation through the IGF-1 axis [33,34], as well as being associated with increased androgen and testosterone levels [42,43,44,45,46]. Diets low in total and saturated fat and high in *n*-3 fatty acids counter this pathway by inhibiting IGF-1 binding and decreasing HSP90 association with the androgen receptor [52]. Consequently, there is increased acetylation of androgen receptors resulting in their degradation, ultimately reducing androgen receptor proteins, as well as the number of androgen receptor-regulated genes.

### 3.3. Dietary Fat Mediation of Cell Proliferation and Angiogenesis

While total dietary fat and saturated fat intake have not been shown to have a direct effect in the cell cycle and angiogenesis, *n*-3 fatty acid intake has been shown to inhibit malignant cell proliferation and angiogenesis [53]. *n*-3 fatty acids work both intrinsically (mitochondrial pathway) and extrinsically (death receptor pathway) to induce apoptosis. Specifically, they can inhibit PI3K activity, which phosphorylates the Akt complex. Phosphorylated Akt regulates a number of downstream factors that can directly affect apoptosis and the cell cycle. Briefly, phosphorylated Akt inhibits caspase 9 and pro-apoptotic proteins Bad and BAK, leading to decreased apoptosis; inhabitation of these pathways via *n*-3 fatty acids ultimately results in increased cell death. Phosphorylated Akt also increases p27 and inactivates NFκB signaling, which can independently and directly halt the cell cycle. Thus, *n*-3 fatty acids may have an important role in inhibiting malignant cell proliferation and angiogenesis.

Total dietary fat and saturated fat may work indirectly to enhance cell proliferation and angiogenesis through the creation of reactive oxygen species (ROS) [54]. ROS generated endogenously and externally are associated with cancer progression by inducing a number of neoplastic transformations. ROS alter the conformational structure of the p53 protein, resulting in changes in protein behaviour and causing a mutated phenotype. These types of p53 mutations are specifically important in prostate cancer progression. Total dietary fat, especially *n*-6 fatty acids, as well as androgens can all serve as oxidants directly increasing oxidative stress and altering a number of transcription factors. Oxidative stress has been shown to be higher in the benign epithelium of men with prostate cancer when compared to men without prostate cancer [54], while Lee *et al.* [55] demonstrated that inactivation of glutathione-s-transferase pi, a pro-oxidant scavenging enzyme, is critical in the development of prostate cancer carcinogenesis. Specifically, dietary fat consumption may contribute to the carcinogenesis of prostate tissue via lipid peroxidation [56], thus resulting in increased oxidative stress.

The relationship between dietary fat, cell proliferation and angiogenesis is less direct than some of the other relationships previously described in this review. There is little evidence to suggest that total and saturated fat intake play any part in malignant cell proliferation and angiogenesis. Dietary fat may be working though secondary pathways, such as ROS generation, to stimulate proliferation and angiogenesis. More research in these areas is needed to elucidate this complex relationship.

## 4. Fatty Acid Type and Its Relationship with Prostate Cancer

While there are many proposed mechanisms through which dietary fat may affect prostate cancer development and progression, much of the literature examining prostate cancer and dietary fat intake lacks a definite conclusion as to the negative impact of dietary fat in prostate cancer. This discrepancy may be, in part, attributed to the diverse physiological effects of different types and distributions of dietary fats; thus, an evaluation of total dietary fat intake may miss important relationships between specific types of dietary fat intake and prostate cancer development. The direct and indirect roles, as well as the interrelationships between saturated fatty acids (SFA), monounsaturated fatty acids (MUFA), polyunsaturated fatty acids (PUFA) and trans fatty acids (TFA) on prostate cancer development and progression need to be elucidated in future work.

There is diverse epidemiological evidence suggesting that SFA intake is a risk factor for prostate cancer. Some studies show increased risk of prostate cancer risk and progression, while others are inconclusive. Animal models suggest that the quality of dietary fat, and, specifically, the PUFA content of dietary fat intake, may be an important prognosticator [57,58]. Long-chain SFA may negatively affect prostate cancer, while short-chain fatty acids may be beneficial. Escobar *et al.* [59] fed rats two isocaloric low-fat diets, in which only 7% of total calorie content was derived from fat. In one diet, fat was derived from lard and the other from linseed oil, which Vereshagin and Novitskaya [60] identified as: 52%–55% α-linolenic acid, 18:3*n*-3; ~7% palmitic acid, 16:0; ~4% stearic acid, 18:0; ~18%–23% oleic acid, 18:1*n*-9; and ~14%–17% linoleic acid, 18:2*n*-6. The lard-derived diet, which was high in palmitic acid and oleic acid, increased prostate weight, testosterone, cell proliferation and androgen receptor expression, compared to the diet rich in α-linolenic acid. These data support the notion that long-chain SFA may have negative effects on various physiological factors in prostate cancer; however, this has yet to be investigated in humans.

The role of MUFA is less clear than the role of SFA in prostate cancer development and progression. The Mediterranean diet, which is rich in oleic acid (18:1*n*-9), was originally thought to reduce the risk of prostate cancer [61]; however, this early evidence remains inconclusive, as studies have demonstrated protective [62], no association [63,64] and negative effects of MUFA on prostate cancer [65,66]. As the Mediterranean diet contains a variety of potential protective agents, such as lycopene-rich tomatoes, fish that are high in *n*-3 fatty acids and low quantities of red meat, it is challenging to pinpoint the role of MUFA.

There is extensive research examining the role of PUFA in prostate cancer. It is reported that *n*-6 fatty acids increase prostate cancer risk, while *n*-3 fatty acids decrease prostate cancer risk [57,58]. Specifically, the anti-cancer benefits of a diet with low *n*-6-to-*n*-3 fatty acid ratios are supported in the literature [67]. This type of diet is in specific contrast to the Western style diet with a 30:1 ratio of *n*-6 to *n*-3 fatty acids [68,69,70,71]. The main mechanisms of action seem to converge on the IGF-1 signaling pathway, leading to prevention or inhibition of malignant growth in prostate cancer cells.

Conversely, a limited number of studies have examined the role of TFA in prostate cancer risk and development. Overall, they appear to suggest an increased risk of prostate cancer with increased serum TFA. Previously, Smith *et al.* [72] reviewed the role of TFA in a number of cancers and identified six studies that examined prostate cancer. Bakker *et al.* [73] conducted an ecological study examining the fatty acid component of adipose tissue in 690 participants across eight European countries and Israel and found no significant relationship between TFA levels and risk of prostate cancer. King *et al.* [74] and Chavarro *et al.* [75] used case-control and nested case-control methodologies, respectively, and demonstrated increased risk in prostate cancer with increased levels of the number of different TFA in both serum phospholipids [74] and whole blood [75]. Food frequency questionnaires have also been used to examine TFA intake and prostate cancer risk and have demonstrated the increased risk of advanced cancer [76] and no relationship [77,78]. More recently, Brasky *et al.* [79] demonstrated an inverse risk between serum TFA and high risk prostate cancer and suggested that the relationship between TFA and prostate cancer risk may be more complicated than earlier hypotheses. Interestingly, Laake *et al.* [80] demonstrated that the source of the TFAs may play a significant role in determining risk, as they demonstrated no association between dietary intake levels of TFA from vegetable sources, but increased risk of prostate cancer when the TFA source is a fish source. This evidence is in the very early stages, where data is associative; thus, more research is warranted to identify potential mechanisms in which prostate cancer is affected by TFA intake, as this relationship appears to be more complex than previously thought.

## 5. Dietary Fat in the Prostate Cancer Survivor

As it is the second most common malignancy diagnosed in men worldwide and because the survival rates are so high [1,2], the number of prostate cancer survivors is constantly increasing. Thus, understanding how manipulating dietary fat may positively influence quality of life in prostate cancer survivorship is important. Another important consideration for prostate cancer survivors is the use of aADT, a common treatment for aggressive prostate cancer, which causes significant loss of skeletal muscle and increases in adipose tissue and has been related to increased risk of cardiovascular disease and diabetes in prostate cancer survivors [7]. However, there is limited data examining dietary fat throughout this stage of the prostate cancer trajectory.

Early reports demonstrate that fat intake, specifically saturated fat intake, may decrease disease-specific survival. Fradet and colleagues [81] followed a group of men diagnosed with prostate cancer for an average of 5.2 years. After controlling for cancer grade, clinical stage, treatment age and total energy intake, men in the lowest tertile of saturated fat intake had a decreased risk of dying from prostate cancer as compared to those in the highest tertile of saturated fat intake (hazards ratio: 3.13; 95% CI: 1.28–7.67). These findings align with the idea that fat intake, specifically saturated fat intake, promotes an environment conducive to prostate cancer growth. While this observational evidence supports the hypothesis that high levels of dietary fat have negative effects on prostate cancer survival, well-designed intervention studies are needed to identify if manipulating dietary fat can have positive effects on survivorship. These interventions should also investigate different sources of dietary fat and their ability to improve quality of life for the prostate cancer survivor by mitigating cancer recurrence, as some literature suggests that plant-based fats may be less harmful than animal-based fats [31,32,59].

Davies and colleagues [82] reviewed the evidence of the effects of low-fat diets on prostate cancer progression and identified five studies that manipulated dietary fat intake in some way to examine the potentially protective effects of these interventions. Ornish *et al.* [83] used a randomized control trial (RCT) design to examine the effect of an entire lifestyle intervention, including low-fat vegan diet supplemented with fish oil and a number of other vitamins and minerals combined with physical activity and stress management techniques, in a group of prostate cancer survivors and examined the effects on the prostate specific antigen (PSA) levels and LNCaP (human prostatic adenocarcinoma) cell growth* in vitro*. This comprehensive lifestyle intervention demonstrated significant improvements for prostate cancer patients* versus* the control group; however, the combined nature of this intervention makes it difficult to discern the individual effects of the lifestyle components (*i.e.*, exercise *vs.* low-fat diet *vs.* stress management techniques). Perhaps the synergistic interactions between these components improve patient outcomes. Two studies have examined the effects of low-fat diets supplemented with flaxseed on prostate cancer outcomes. Demark-Wahnefried *et al.* [84] demonstrated decreased proliferation rates in the men supplemented with flaxseed and that the low-fat diet group had significantly reduced serum cholesterol levels following ~30 days of supplementation. Heymach *et al.* [85] demonstrated that, as compared to the control arm, a low-fat diet, a flaxseed-supplemented diet and a low-fat diet with a flaxseed supplementation for 30 days had each decreased a number of angiogenic factors, though the results were greatest in the low-fat diet alone group. They speculate that the NFκB pathway may be regulating this response. A review by Hori *et al.* [86] supports the hypothesis suggested by the flax-supplemented diet that *n*-3 fatty acid may be beneficial for prostate cancer patients. Like Ornish *et al.* [83], Aronson *et al.* [87] used an RCT design to look at the effects of a four-week low-fat diet intervention as compared to a traditional Western diet on the effects of LNCaP cell growth. Serum for the low-fat diet group decreased the growth of the LNCaP cells as compared to the serum from the men on the Westernized diet. While there appears to be some evidence as to the protective effect of a low-fat diet for prostate cancer survivors, more research is warranted to better elucidate the specific components of these lifestyle interventions that will be most effective for prostate cancer patients.

### Androgen Deprivation Therapy and Dietary Fat

ADT is a common treatment for prostate cancer patients; however, its use can have negative consequences for prostate cancer survivors. Specifically, patients undergoing ADT lose skeletal muscle mass and gain fat mass [88]. These changes are associated with increased risk of cardiovascular disease and diabetes [7]. Because of the associated changes in body composition and risk of comorbidities in survivorship, dietary intervention may be useful for prostate cancer patients receiving ADT. However, these considerations are beyond the scope of this review. Saylor and Smith [89] suggest lifestyle intervention, including low-fat diet and increased physical activity and weight control, may be beneficial in the prevention of these comorbidities in men receiving ADT and that future investigations are justified.

## 6. Conclusions

The aim of this paper was to examine dietary fat throughout the prostate cancer trajectory, including risk, development and survivorship. In most cases, there is limited evidence linking dietary fat and prostate cancer; however, some trends do emerge. Dietary fat, and, in particular, high intake of animal and saturated fats, may be associated with prostate cancer risk. The IGF/Akt signaling pathway appears to be the key signaling pathway moderating malignant cell growth and changes in androgen receptor signaling. The type of fat consumed may mediate the relationship between dietary fat and prostate cancer. Saturated fat and TFA have been negatively associated with prostate cancer development, while PUFA may have a protective effect, though these relationships remain tenuous. For prostate cancer survivors, a diet low in fat and particularly low in saturated fat may be beneficial, as it may reduce tumor angiogenesis and cancer recurrence. Integrating research throughout the prostate cancer trajectory may provide new insights into the relationship between dietary fat intake and prostate cancer, as many of the same pathways are implicated throughout the trajectory. In conclusion, preliminary evidence suggests diets low in fat may be beneficial at any point in the prostate cancer trajectory; however, much more research is needed to elucidate the complex relationships that exist between dietary fat and prostate cancer biology.
